# High Fat Diet with a High Monounsaturated Fatty Acid and Polyunsaturated/Saturated Fatty Acid Ratio Suppresses Body Fat Accumulation and Weight Gain in Obese Hamsters

**DOI:** 10.3390/nu9101148

**Published:** 2017-10-19

**Authors:** Suh-Ching Yang, Shyh-Hsiang Lin, Jung-Su Chang, Yi-Wen Chien

**Affiliations:** 1 School of Nutrition and Health Sciences, Taipei Medical University, Taipei 11031, Taiwan; sokei@tmu.edu.tw (S.-C.Y.); lin5611@tmu.edu.tw (S.-H.L.); susanchang@tmu.edu.tw (J.-S.C.); 2 Research Center of Geriatric Nutrition, College of Nutrition, Taipei Medical University, Taipei 11031, Taiwan; 3 Graduate Institute of Metabolism and Obesity Sciences, Taipei Medical University, Taipei 11031, Taiwan

**Keywords:** diet-induced obesity, monounsaturated fatty acids, acyl-CoA oxidase, fat accumulation

## Abstract

The aim of this study was to investigate the effect of a high fat diet with experimental oil consisting of 60% MUFAs (monounsaturated fatty acids) with a P/S ratio of 5 on fat deposition and lipid metabolism in obese hamsters. Hamsters were randomly assigned to a control group and a diet-induced obesity group for nine weeks. Then an additional eight-week experimental period began, during which obese hamsters were randomly divided into three groups and fed different amounts of the experimental oil mixture in their diets as follows: 5%, 15%, and 20% *w*/*w* (OB-M5, OB-M15, and OB-M20 groups, respectively). The results showed that the OB-M_15_ and OB-M_20_ groups had significantly lower blood cholesterol and higher insulin levels. Compared to the control group, the three obese groups exhibited higher hepatic fatty acid synthase activity; however, the acyl-CoA oxidase activities were also enhanced. Although dietary fat content differed, there were no differences in energy intake, final body weights, and epididymal fat weights among the four groups. These results suggest that regardless of whether the specimens had a high fat intake or not, dietary fat containing high MUFAs with a high P/S ratio had beneficial effects on maintaining blood lipid profiles and may not result in body fat accumulation in obese hamsters, possibly by promoting lipolytic enzyme activities.

## 1. Introduction

Obesity is considered to be a major risk factor for metabolic syndrome and some types of cancer [1,2]. The most common causes are excessive food intake, a lack of exercise, and a genetic predisposition [3]. A diet rich in fat (more than 30% of energy) induces the development of obesity, because dietary fat intake provides a higher dietary energy density, and it is also difficult to promote fat oxidation as a source of energy [4]. Achieving a healthy eating pattern may require adjustments in food and dietary oils containing different types and amounts of fatty acids. Low-fat diets, especially low-saturated-fat diets, may be an effective method of supporting a healthy body weight and improving obesity-related complications [5,6]. To promote health and prevent diet-related chronic diseases, the following recommendations for fat intake were established: saturated fatty acids (SFAs) should be <10% of energy and trans fatty acids <1% of energy [7,8]. But, several dietary cooking oils affect body weight gains and their use remains controversial.

Different types of fatty acids have different oxidation and deposition rates that may contribute to fat accumulation and weight gain [9,10,11]. Those reports showed the positive effects of monounsaturated fatty acids (MUFAs) on weight control by increasing postprandial fat oxidation and diet-induced thermogenesis compared to SFAs [9,10] or n-3 polyunsaturated fatty acids (PUFAs; flaxseed oil rich in linolenic acid) [11]. By measuring isotope-labeled fatty acids, some studies found that the oxidation of MUFAs was greater than that of n-6 PUFAs in an animal model [12], with similar results in humans [13]. One recent study reported that unsaturated fatty acids induced higher diet-induced thermogenesis and fat oxidation following a high-fat meal [14]. A high-MUFA diet, however, consisting of more than 38% of total energy, had no beneficial effects on insulin sensitivity in healthy subjects [15]. Moreover, human studies showed that a diet with a high polyunsaturated to saturated fatty acid (P/S) ratio also increased postprandial fat oxidation [16,17]. We previously demonstrated that high-MUFA oil with a high P/S ratio (consisting of 60% MUFAs of the total fatty acids with a high P/S ratio of 5) may prevent high-fat diet-induced body weight and body fat accumulation by decreasing adipose peroxisome proliferator-activated receptor (PPAR)γ and lipoprotein lipase (LPL) messenger (m)RNA expressions and improving hepatic lipolytic enzyme activities [18,19].

Fatty acids regulate lipogenesis through various transcription factors and nuclear receptors, including PPARs, hepatocyte nuclear factor (HNF)-4α, liver X receptor (LXR), and sterol response element-binding proteins (SREBPs) [20]. There are three PPAR isoforms, termed PPARα, PPARγ, and PPARβ/δ [21], that participate in fatty acid transport and oxidation accompanied by the regulation of fat mass [22]. PPARγ is highly expressed in white adipose tissue, and its activation plays a key role in adipocyte development and differentiation [23]. PPARγ activation mediates the expressions of several target genes implicated in adipose tissue accumulation, such as LPL and hormone-sensitive lipase (HSL) [24]. LPL is located on the surface of endothelial cells and is an enzyme responsible for the hydrolysis of blood triglyceride (TG)-derived lipoproteins, including chylomicrons and very-low-density lipoproteins [25]. These hydrolyzed fatty acids directly enter peripheral tissues and white adipose tissue. In contrast, HSL is a key enzyme that catalyzes the hydrolysis of diacylglycerol to monoacylglycerol in adipose tissues, and its high activity attenuates TG accumulation in adipocytes [26]. SREBP-1c regulates hepatic lipid metabolism and insulin-induced lipogenesis, and its target genes include fatty acid synthase (FAS), acetyl-CoA carboxylase (ACC), and the low-density lipoprotein receptor [27]. In the liver, a PUFA-enriched diet inhibits hepatic fatty acid synthesis by suppressing the transcription of the SREBP-1c gene compared to a MUFA-enriched diet (using olive oil) [28,29,30].

A previous study in obese men showed that a moderate-fat (33% of energy) diet resulted in favorable changes in serum lipid profiles and weight loss [31]. Our recent study demonstrated that different amounts of high-MUFA oil with a high P/S ratio prevent body weight gain and body fat accumulation by increasing hepatic carnitine palmitoyltransferase-1 (CPT-I) activities with a dose-dependent effect [32]. To further investigate whether high-MUFAs with a high P/S ratio oil can retard the progression of high-fat diet-induced obesity, we used hamsters with diet-induced obesity to study the effects of three different quantities (5%, 15%, and 20% *w*/*w*) of dietary fat intake on the fat accumulation mechanism by measuring hepatic enzyme activities and adipose gene expressions.

## 2. Materials and Methods

### 2.1. Animals and the Study Design

**Figure 1:**
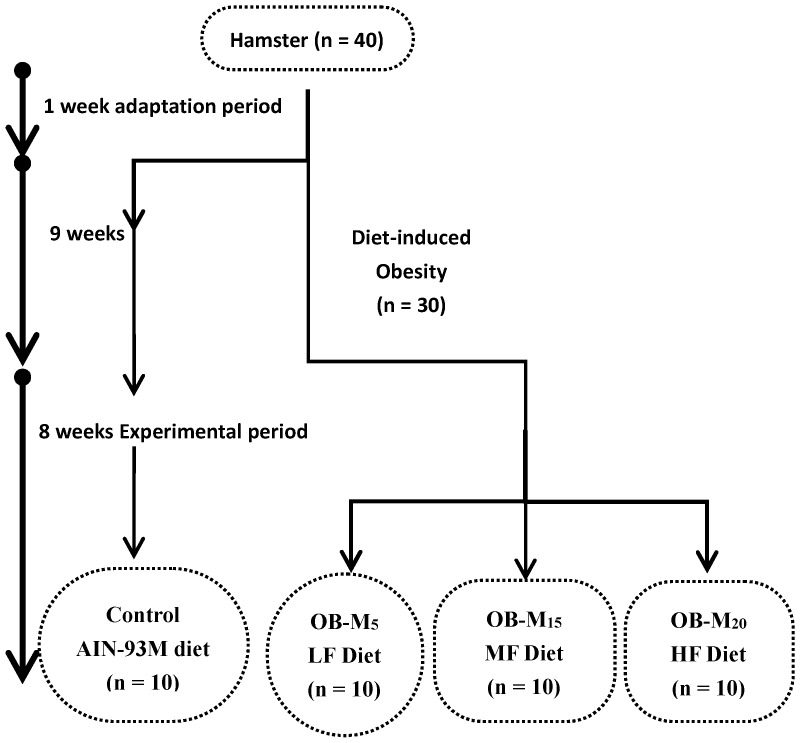
The schema of the experimental protocol.

Golden Syrian hamsters were purchased from the National Laboratory Animal Center (Taipei, Taiwan) and kept in an air-conditioned room with a 12-h light-dark cycle at 22 ± 2 °C and 65% ± 5% relative humidity. All animal experimental procedures followed published guidelines approved by the Institutional Animal Care and Use Committee of Taipei Medical University, Taipei, Taiwan (LAC-2013-0240). For a one-week acclimation period, 32 male seven-week-old hamsters were given free access to regular rodent chow and water *ad libitum*. Hamsters were randomly assigned to a control group (*n* = 8) fed a low-fat diet (5% *w*/*w* soybean oil, 3.85 kcal/g) and a diet-induced obesity group (DIO, *n* = 24) fed a high-fat diet (35% *w*/*w* soybean oil, 5 kcal/g, 52% of energy), according to the AIN-93M formulation [33] and modification, for nine weeks. Then an additional eight-week experimental period began, in which hamsters with diet-induced obesity were randomly divided into three groups (*n* = 8 per group) and fed different amounts of the experimental oil mixture in their diets as follows: 5%, 15%, and 20% *w*/*w* (OB-M_5_, OB-M_15_, and OB-M_20_ groups, respectively) (Figure 1). The experimental oil was a mixture of canola oil and soybean oil. The SFA/MUFA/PUFA ratio of the experimental oil was 1/7.1/4.7 with an omega-6/omega-3 ratio of 3.58. The control group was still fed a low-fat diet as described above (the SFA/MUFA/PUFA ratio was 1/1.6/4.1 with an omega-6/omega-3 ratio of 8.39), as previously described [32]. All hamsters had *ad libitum* access to their food and water. The composition of the experimental diets is shown in Table 1. Food intake was measured daily, and body weights were measured weekly. At the end of the experimental period, hamsters were starved for 12 h, and anesthetized with an intraperitoneal injection of an equal-volume mixture of 1 mL/kg Zoletil 50 (Virbac, Carros, France) and Rompun 20 (Bayer Korea, Seoul, Korea). Blood samples were drawn by cardiac puncture and transferred into tubes containing ethylenediaminetetraacetic acid (EDTA), and plasma was immediately collected following centrifugation at 3500 rpm for 15 min at 4 °C. Liver and epididymal white adipose tissues were dissected, weighed, and quickly frozen in liquid nitrogen. Epididymal fat was collected from the surroundings of the epididymis and testis. Retroperitoneal fat was collected from the surroundings of the kidney to the abdominal wall. All samples were stored at −80 °C before being analyzed.

### 2.2. Fatty acid Composition Analysis

We purchased canola oil and soybean oil from local supermarkets and analyzed the fatty acid composition by gas chromatography (GC) using heptadecanoic acid (C17:0) as an internal standard. According to the method of Morisson and Smith [33], fatty acids were converted to methyl esters with 14% boron trifluoride in methanol at 95 °C for 30 min and then separated and quantified using a 30-m-long, 0.32-mm-ID, 0.32-µm-df capillary column (Restek, Bellefonte, PA, USA) and a flame ionization detector on a capillary gas chromatograph (Thermo Finnigan Trace GC, Milan, Italy). Table 2 shows the fatty acid composition of the different diets.

### 2.3. Plasma Measurements

Plasma total cholesterol, TG, high-density lipoprotein cholesterol (HDL-C), low-density lipoprotein cholesterol (LDL-C), nonesterified fatty acids, and fasting glucose concentrations were analyzed by enzymatic colorimetric assays using commercial enzyme kits (Randox Laboratory, Crumlin, Northland, UK). Plasma insulin concentrations were determined using a commercially enzyme-linked immunosorbent assay (ELISA) kits (Mercodia AB, Uppsala, Sweden). Plasma leptin and adiponectin concentrations were measured using a hamster leptin immunoassay and a hamster adiponectin ELISA kit, respectively, (Blue Gene Biotech, Shanghai, China) using a VERSAmax microplate reader (Molecular Devices, Sunnyvale, CA, USA).

### 2.4. Hepatic Lipid Contents and Enzyme Activities

Hepatic lipids were extracted according to a method described by Folch et al. [34] and hepatic total cholesterol and TG contents were measured using commercial enzyme kits (Randox Laboratory, Antrim, UK) and expressed as milligrams per gram of liver. Hepatic nonesterified fatty acid levels were measured using commercial enzyme kits (Cayman Chemical, Ann Arbor, MI, USA) and expressed as millimoles per gram of liver.

The activities of hepatic lipogenic enzymes, including fatty acid synthase (FAS) and acetyl-CoA carboxylase (ACC), and lipolysis enzymes, such as acyl-CoA oxidase (ACO) and carnitine palmitoyltransferase-1 (CPT-I), were assessed according to previously described protocols [18].

### 2.5. Adipose Tissue LPL Enzyme Assay

A portion of epididymal adipose tissue was homogenized in assay buffer and centrifuged at 13,000× *g* for 10 min using a commercial lipase activity colorimetric assay kit (BioVision, Milpitas, CA, USA) according to the manufacturer’s instructions.

### 2.6. Real-Time Quantitative Polymerase Chain Reaction (qPCR)

Total RNA from epididymal white adipose tissues was extracted using TRIzol (Invitrogen, Carlsbad, CA, USA), according to the manufacturer’s procedures, and complementary (c)DNA was synthesized from 3 μg of RNA in a 20-μL reaction volume using a First Strand cDNA Synthesis Kit with oligo(dT)18 primers (Applied Biosystems, Foster City, CA, USA) using the following program: 65 °C for 5 min, 42 °C for 60 min, 70 °C for 5 min, and a 4 °C hold in an Applied Biosystems Veriti 96 Well Thermal Cycler. A real-time PCR was performed using 100 ng cDNA as the template with SYBR Green PCR Master Mix (Applied Biosystems, Foster, CA, USA) following the protocol as previously described [32] on a 7300 Real-Time PCR System (Applied Biosystems, Foster, CA, USA). To evaluate gene expressions, β-actin was used as a reference gene to normalize the expressions of three target genes including PPARγ, LPL, and HSL. The assay included a no-template control to detect reagent contamination and additional DNA products. The primer information was reported as previously described [32].

### 2.7. Histological Analyses

Liver tissue was excised, flushed with PBS, and fixed in 10% formaldehyde for 1hr before being embedded in paraffin. Samples were stained with haematoxylin and eosin (H & E) with standard protocol. Histological images were obtained on an Olympus IX71 microscope with an Olympus DP70 camera using Olympus DP controller software.

### 2.8. Statistical Analysis

Data are presented as the mean ± standard deviation (SD). We used an unpaired *t*-test to compare the differences between the obesity and control groups through the DIO period. To evaluate the significance of the four groups, a one-way analysis of variance (ANOVA) with a Fisher’s least significant difference (LSD) test was used in SPSS vers. 18.0 (SPSS, Chicago, IL, USA). Adipose tissue gene expressions among groups were statistically analyzed with the Kolmogorov-Smirnov test. Differences were considered statistically significant at *p* < 0.05.

## 3. Results

### 3.1. Body and Tissues Weights, Weight Gain, and Energy Consumption

The initial body weight at the beginning of the study was 84.2 ± 1.8 g. After nine weeks of the DIO period, the obesity group fed a fat-rich diet had gained more weight than the control group (145.9 ± 2.3 vs. 135.8 ± 1.5 g, *p* < 0.05). In the eight-week experimental period, the control group and three DIO groups had similar energy intakes, while the OB-M_15_ and OB-M_20_ groups had a significantly decreased food intake compared to the control group. The OB-M_5_ group had a significantly lower weight gain compared to the control group, but no difference in weight gain was found among the OB-M_15_, OB-M_20_, and control groups. No significant change in epididymal or retroperitoneal fat weights was observed among the OB-M_5_, OB-M_15_, and OB-M_20_ groups (Table 3).

### 3.2. Plasma Parameter Concentrations and Hepatic Lipid Content

The OB-M_15_ and OB-M_20_ groups had significantly lower plasma total cholesterol and HDL-C levels than the OB-M_5_ group, and significantly lower plasma LDL-C levels and higher plasma insulin concentrations than the control group. The OB-M_15_ group had the lowest plasma HDL-C level among all groups (*p* < 0.05). No differences in plasma TG, free fatty acids (FFA), or glucose concentrations were observed (Table 4). Hepatic free fatty acid contents were highest in the OB-M_20_ group, modest in the OB-M_15_ group, mild in the OB-M_5_ group, and lowest in the control group (*p* < 0.05, Table 5). No differences in liver total cholesterol or TG contents were found. Plasma leptin and adiponectin concentrations were unaffected among the four groups.

### 3.3. Hepatic Lipogenic and Lipolysis Enzyme Activities

Hepatic FAS activities were higher in obese hamsters among the three groups than in the control group. The OB-M_20_ group exhibited a significant decrease in hepatic ACC activities compared to the OB-M_15_ group. No difference in hepatic ACC activities was found between the OB-M_5_ and control groups. In the analysis of lipolysis enzyme activities, the three obese groups fed the experimental oil had significantly improved hepatic ACO activities compared to the control group. Hepatic ACO activities were greater in the OB-M_15_ group than in both the OB-M_5_ and OB-M_20_ groups, and were lowest in the control group (*p* < 0.05). No differences in hepatic CPT-I activities were observed among the four groups (Table 4).

### 3.4. Adipose Tissue LPL Activities

LPL activities in epididymal fat and retroperitoneal fat exhibited no differences among the four groups (Table 4).

#### Liver Histological Analyses

**Figure 2:**
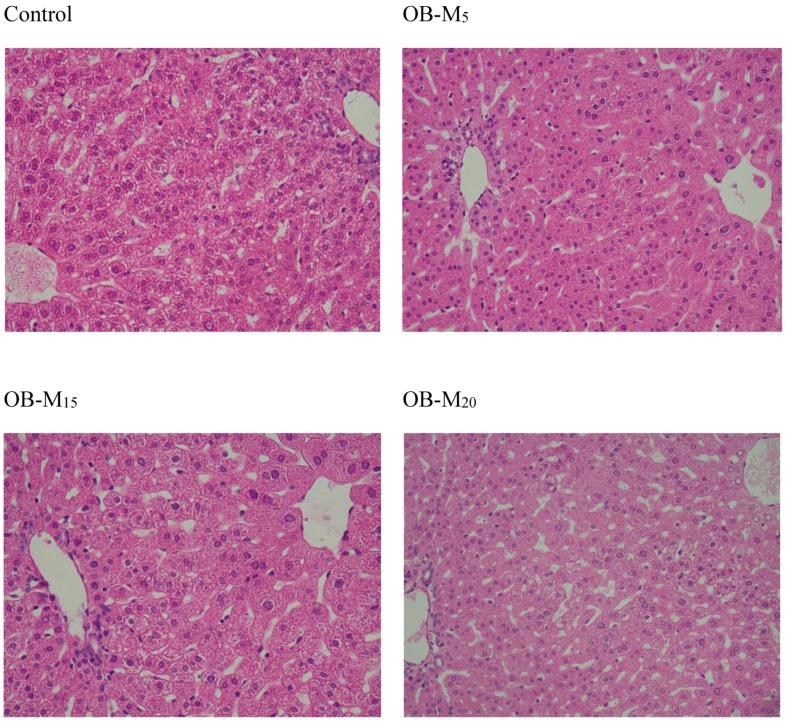
Effects of the experimental oil on the haematoxylin and eosin staining of histologically sectioned liver tissues in hamsters (200X). Control, 5% *w*/*w* soybean oil; OB-M_5_, 5% *w*/*w* of experimental oil mix; OB-M_15_, 15% *w*/*w* of experimental oil mix; OB-M_20_, 20% *w*/*w* experimental oil mix.

The histologic images by H & E staining of the livers in the control group showed normal liver architecture. Also, there was no accumulation of lipid droplets in the livers of the OB-M_5_, OB-M_15_, and OB-M_20_ groups (Figure 2).

### 3.5. Lipid Metabolism-Related Protein mRNA Expression in Adipose Tissue

**Figure 3:**
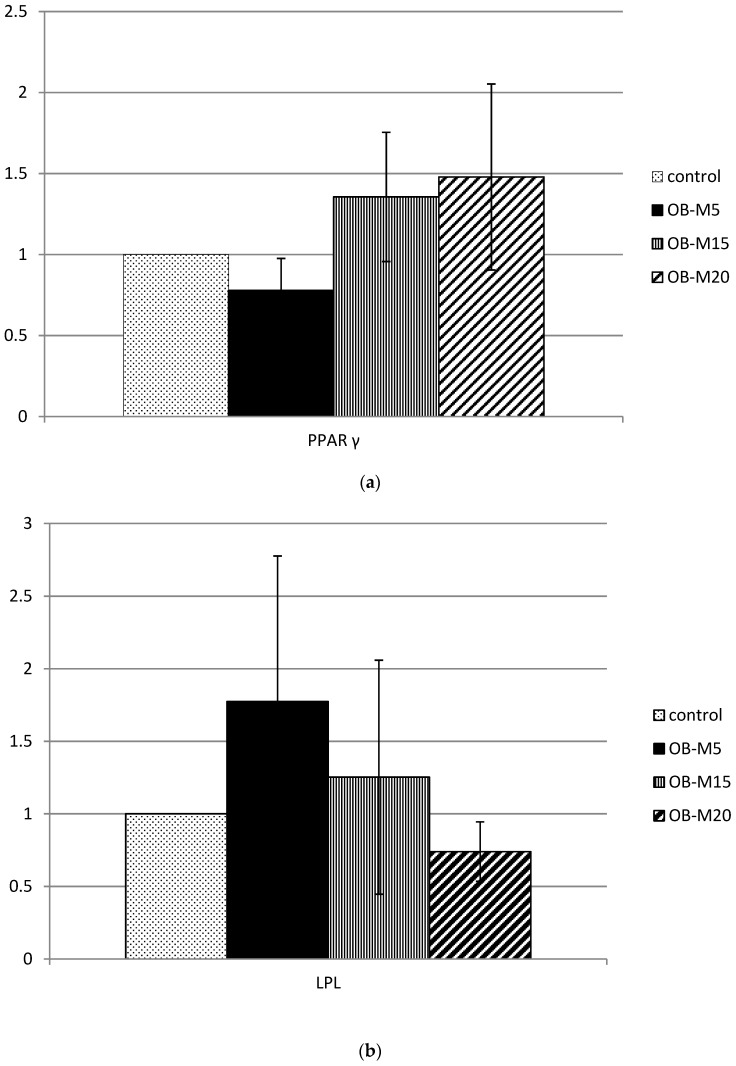

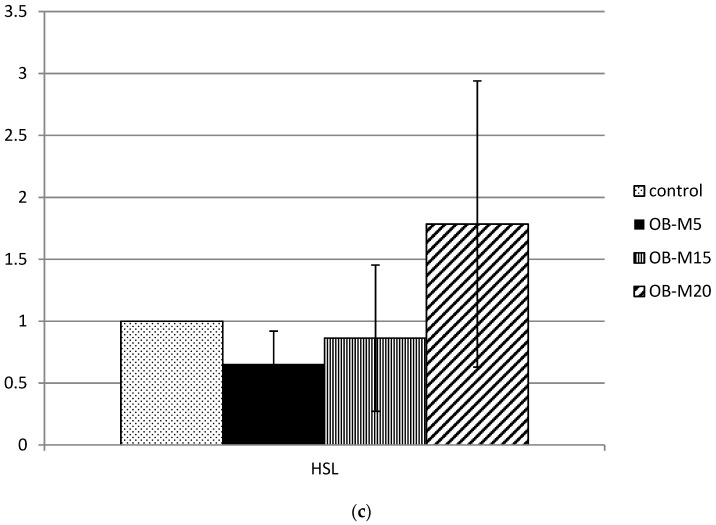
Real-time quantitative PCR analysis of peroxisome proliferator activator receptor (PPAR)γ, lipoprotein lipase (LPL), and hormone-sensitive lipase (HSL) mRNA levels in epididymal white adipose tissues of hamsters with diet-induced obesity fed the experimental oil for eight weeks. Values are presented as the mean ± SEM (*n* = 7). PPARγ mRNA levels were normalized to glyceraldehyde-3-phosphate dehydrogenase (GAPDH) mRNA levels. LPL and HSL mRNA levels were normalized to β-actin mRNA levels. The graph represents mRNA levels of PPARγ (**a**); LPL (**b**); and HSL (**c**) relative to the control group. Control, 5% *w*/*w* soybean oil; OB-M_5_, 5% *w*/*w* of experimental oil mix; OB-M_15_, 15% *w*/*w* of experimental oil mix; OB-M_20_, 20% *w*/*w* experimental oil mix.

There was no difference in the mRNA expressions of PPARγ, LPL, or HSL among the four groups (Figure 3).

## 4. Discussion

In this study, we found that DIO hamsters fed 15% and 20% *w*/*w* of dietary fat intake, representing approximately 31% and 39.1% of the total energy intake, respectively, exhibited no significant difference in fat mass or weight gain compared to control hamsters fed a low-fat diet (5% *w*/*w* oil), representing about 11.7% of total energy from fat, under ad libitum feeding conditions in response to an isocaloric intake. Only 5% *w*/*w* of experimental oil feeding in DIO hamsters (OB-M_5_ group) produced much less weight gain compared to 5% soybean oil (*w*/*w*) feeding in control hamsters, which was similar to the results of our previous study [18]. The main reason we chose Golden Syrian hamsters as the experimental model is that fat-fed hamsters exhibit dietary obesity stemming from a decrease in energy expenditure (diet-induced thermogenesis) [35], not overeating, which resembles blunted diet-induced thermogenesis in obese humans [36,37]. Hall et al. reported that fat restriction led to greater body fat loss than carbohydrate restriction in obese men [38]. The interesting results presented herein imply that the effects of high-fat diets using oil consisting of 60% MUFAs with a high P/S ratio of 5 on fat accumulation were similar to a low-fat weight control diet under an isoenergetic intake, suggesting that the use of this oil in the diet to decrease fat deposition might be a meal replacement for dietary fat restriction to lose weight.

This study further demonstrated that a high amount of oil consisting of 60% MUFAs with a high P/S ratio of 5 in diets reduced body weight gain and decreased fat accumulation in DIO hamsters by enhancing hepatic ACO activities (the rate-limiting enzyme of peroxisomal β-oxidation) with no enhancement of PPARγ or LPL mRNA expressions or LPL activities. It is well known that high-fat diets lead to adipose PPARγ activation which mediates adipogenesis [23]. The influences of types of fatty acids on PPARγ were reported by Xu et al.; they found that unsaturated fatty acids interacted more efficiently with PPARγ, and oleic acid interacted much more efficiently with PPARγ than linoleic acid (C18:2) and alpha-linolenic (C18:3), but saturated fatty acids failed to bind well with any of the PPARs [39]. In this study, qPCR results revealed that the unaltered expression of PPARγ was observed in obese hamsters fed three different amounts of the experimental oil, suggesting that the type of fatty acid-mediated PPARγ expression was more dominant than the amount of dietary fat.

Both leptin and adiponectin as adipocyte-derived hormones are associated with energy expenditures [40,41]. Animal and cellular studies by Bueno et al. demonstrated that both the serum adiponectin concentration and adiponectin gene expression in epididymal adipose tissues were reduced in mice chronically fed high-fat diets enriched with soybean oil (n-6 PUFA) and coconut oil (SFA). The same reduction in levels of adiponectin gene expression was seen in 3T3-L1 cells treated with palmitic, linoleic, EPA, and DHA acids [42]. MUFA-rich high-fat diets (Mediterranean diet, 38% of energy) decreased serum leptin levels, but fasting and postprandial serum adiponectin and resistin remained unchanged in obese type 2 diabetic patients [43]. In addition, Kennedy et al. demonstrated that adiponectin increased in response to a high-energy meal independent of amounts of dietary fat in lean, healthy men [41]. In this regard, potential reasons for no alteration in plasma adiponectin and leptin concentrations in DIO hamsters fed different amounts of dietary fat may be related to isocaloric intake in the present study.

In general, triacylglycerol from adipose tissues is hydrolytically cleaved by lipolysis to generate glycerol and free fatty acids that are released into the blood for subsequent uptake by other organs as energy substrates. Alternatively, glycerol is transferred to the principal site for reuse in TG synthesis in the liver. Taken together with blood and hepatic lipid profiles, lipid contents, excluding hepatic free fatty acid, were not influenced when the total fat intake increased (OB-M_15_ and OB-M_20_ vs. the control and OB-M_5_). Other molecular mechanisms of moderate lipid profiles of obese hamsters fed the experimental oil mixture may be that n-6 PUFAs modulate the downregulation of proprotein convertase subtilisin/kexin type 9 (PCSK9, a hepatic LDL-receptor regulator) [44] and upregulation of hepatic scavenger receptor B1 (SR-BI) [45]. Characteristics of the fatty acid composition of the experimental oil used here were a combination of high MUFAs and a higher P/S ratio, and the beneficial effects of <10% SFAs on lipid concentrations cannot be ignored [8,46]. Data from Lopez et al. confirmed that MUFAs in high-fat meals are superior to SFAs in reducing the dysregulation of postprandial lipid metabolism and insulin intolerance [46]. Additionally, Kruse et al. reported that rapeseed/canola oil in the diet improves serum lipids and basal inflammation in adipose tissue compared to olive oil in obese men [47]. The failure to observe excessive blood and hepatic lipid contents by the high-fat diets in this study may have been due to the diets predominantly containing unsaturated fatty acids. Since it appeared that no difference in liver weight, fat weight, and lipid droplets accumulation in the liver was found, the possible explanation might be that there were differences in the lean body mass among four groups in response to different oxidation and deposition rates of different types and amounts of fatty acids.

High-fat diets not only elevated the hepatic free fatty acid levels associated with non-alcoholic fatty liver disease through cellular oxidative stress, but also led to insulin resistance related to hepatic tumor necrosis factor (TNF)-α and interleukin-6 [48,49]. Here, possible explanations for the high hepatic free fatty acid content in the OB-M_20_ group were enhanced HSL (a principal regulator of FFA release from adipose tissues) mRNA expression and plasma insulin concentrations [50]. Plasma insulin levels, generally known to promote lipogenesis and its target genes, including FAS and ACC, were raised in both the OB-M_15_ and OB-M_20_ groups, whereas elevated hepatic ACC activities were observed in the OB-M_15_ but lower activities were observed in the OB-M_20_ group. We believe that increases in plasma insulin concentrations and hepatic FAS activities among the three obese groups were associated with the development of obesity via the consumption of long-term high-fat diets in the present study. In addition, feeding a high amount of experimental oil in obese hamsters improved hepatic ACO activities leading to fat loss. Furthermore, lipocalin is a pro-inflammatory adipokine up-regulated in obesity and positively correlated with the un favourable lipid profiles. Lipocalin 2 is an adipokine with potential importance in insulin resistance associated with obesity. We will be including it in our next set of experiments.

Relationships between adipose HSL mRNA expression and types of fatty acids were reported by van Hees et al., in which HSL mRNA and protein expressions were not affected by the dietary fat composition [51], in agreement with our previous study that both MUFAs and the P/S ratio had minimal effects on adipose HSL mRNA expression [18]. The dietary fat quantity, rather than the types, may modulate HSL mRNA and protein expressions in an obese status [51], although HSL mRNA expression in the OB-M_20_ group exhibited no significant increase compared to the OB-M_5_ or control groups. Despite the lack of differences in gene expressions in white adipose tissues in this report, more studies are required to further understand adipose tissue gene expressions in response to various types and amounts of fatty acids.

Over the last few decades, the nutritional changes associated with the increased consumption of LA-rich vegetable oils in the Western diet show that the ratio of omega-6/omega-3 fatty acids has increased to be within the range of 10:1 to 20:1. Then, the omega-6/omega-3 fatty acids ratio are also considered to be associated with obesity, and optimal dietary intakes of the omega-6/omega-3 ratio should be around 1–4:1 [52,53]. Indeed, the omega-6/omega-3 ratio of the experimental oil was lower than that of soybean oil. That was an alternative possibility of the discriminative effects of dietary cooking oils on the regulation of body fat deposition. However, Enos et al. reported that reducing the omega-6/omega-3 ratio using alpha-linolenic acid is not an effective therapy for attenuating obesity development [54]. In this study, the experimental oil and soybean oil had a similar alpha-linolenic acid content, with values of 7.95% and 6.52%, respectively. Thus, dietary cooking oil containing higher MUFA percentages accompanied by an increasing P/S ratio was designed and it has been proven to much more efficiently reduce fat deposition than oil containing lower MUFA percentages with a lower P/S ratio [18,19,32].

One of the limitations in this study was sample size, which is this reason for a large SEM (the standard error of the mean) in the RT-PCR data of the OB-M20 group because of the existence of the variation among the experimental animals. Other limitations were that the representative white adipose tissues, epididymal and retroperitoneal fat, were dissected and weighed, but within the various white adipose tissues of male mice, including murine intra-abdominal pads (e.g., epididymal, retroperitoneal, mesenteric fat) and subcutaneous pads (e.g., inguinal fat), the lean body mass was unknown.

## 5. Conclusions

In conclusion, these results suggest that dietary patterns using both low and high amounts of oil consisting of 60% MUFAs with a high P/S ratio of 5 revealed benefits in terms of preventing fat accumulation and normalizing blood lipid profiles in obese hamsters. Using this oil in diets may potentially be advantageous for body composition and weight management, but low-fat diets are generally recommended.

## Figures and Tables

**Table 1:** Composition of the experimental diets.

Component (g/100 g of the Diet)	C	OB-M_5_	OB-M_15_	OB-M_20_
Casein	14	14	14	14
Corn starch	61.07	61.07	51.07	46.07
Sucrose	10	10	10	10
Cellulose	5	5	5	5
Soybean oil	5	0	0	0
Experimental oil	0	5	15	20
AIN-93 Mineral mix ^†^	3.5	3.5	3.5	3.5
AIN-93 Vitamin mix ^‡^	1	1	1	1
L-cysteine	0.18	0.18	0.18	0.18
Choline bitartrate	0.25	0.25	0.25	0.25
Tert-butylhydroquinone	0.001	0.001	0.001	0.001
Calorie/g				
CHO% of energy				
Protein% of energy				
Fat% of energy	11.7	11.7	31	39.1

^†^ The mineral mixture contained the following (mg/g): calcium phosphate dibasic, 500; sodium chloride, 74; potassium sulfate, 52; potassium citrate monohydrate, 20; magnesium oxide, 24; manganese carbonate, 3.5; ferric citrate, 6; zinc carbonate, 1.6; curpric carbonate, 0.3; potassium iodate, 0.01; sodium selenite, 0.01; and chromium potassium sulfate, 0.55; ^‡^ The vitamin mixture contained the following (mg/g): thiamin hydrochloride, 0.6; riboflavin, 0.6; pyridoxine hydrochloride, 0.7; nicotinic acid, 3; calcium pantothenate, 1.6; D-biotin, 0.05; cyanocobalamin, 0.001; retinyl palmitate, 1.6; dl-α-tocopherol acetate, 20; cholecalciferol, 0.25; and menaquinone, 0.005. C_5_, 5% *w*/*w* soybean oil; OB-M_5_, 5% *w*/*w* experimental oil mix; OB-M_15_, 15% *w*/*w* experimental oil mix; OB-M_20_, 20% *w*/*w* experimental oil mix.

**Table 2:** The fatty acid composition of the diets.

Fatty Acid %	Soybean Oil	Experimental Mix Oil
C14-0S	0.12	0.03
C16-0S	11.25	5.76
C18-0S	3.48	1.99
C16-1n7	0.11	0.24
C18-1n9	23.46	54.11
C20-1n9	0.13	0.97
C18-2n6	54.87	26.32
C20-2n6	0.05	2.52
C18-3n3	6.52	7.95
C20-5n3	0.02	0.08
C22-6n3	0	0.03
Total SFA	14.84	7.78
Total MUFA	23.70	55.32
Total PUFA	61.46	36.90
P/S ratio	4.1	5.0
S/M/P proportion	1:1.6:4.1	1:7.1:4.7
n-6/n-3 ratio	8.39	3.58

**Table 3:** Body and tissue weights, weight gain, and energy consumption of the eight-week experimental period.

Variables	C	OB-M_5_	OB-M_15_	OB-M_20_
Initial body weight (g)	135.8 ± 1.5 ^b^	145.7 ± 5.1 ^a^	146.1 ± 3.5 ^a^	146.0 ± 3.8 ^a^
Final body weight (g)	153.5 ± 4.4	153.2 ± 6.1	161.0 ± 4.0	164.5 ± 2.3
Liver weight (g)	5.1 ± 0.3	5.5 ± 0.3	5.4 ± 0.2	5.4 ± 0.2
Epididymal fat weight (g)	2.7 ± 0.2	2.9 ± 0.3	3.1 ± 0.2	2.8 ± 0.2
Retroperitoneal fat weight (g)	0.9 ± 0.0	1.0 ± 0.1	0.9 ± 0.0	1.0 ± 0.0
Weight gain (g)	16.2 ± 3.1 ^a^	10.6 ± 4.3 ^b^	14.9 ± 8.1 ^a^	18.3 ± 1.0 ^a^
Food intake (g/day)	8.6 ± 0.3 ^a^	8.9 ± 0.2 ^a^	7.6 ± 0.1 ^b^	7.5 ± 0.2 ^b^
Energy intake (kcal/day)	33.0 ± 1.0	34.3 ± 0.8	33.2 ± 0.6	34.6 ± 0.9
Feed efficiency (%)	0.20 ± 0.04	0.10 ± 0.04	0.10 ± 0.05	0.10 ± 0.01

Data are expressed as the mean ± SD (*n* = 8/group). Feed efficiency = [weight gain (kg)/food intake (g)] × 100%. C, 5% *w*/*w* soybean oil; OB-M_5_, 5% *w*/*w* experimental oil mix; OB-M_15_, 15% *w*/*w* experimental oil mix; OB-M_20_, 20% *w*/*w* experimental oil mix. ^a,b^ Values with different superscripts significantly differ (*p* < 0.05).

**Table 4:** Plasma parameters of hamsters with diet-induced obesity fed the experimental oil for eight weeks.

Variables	C	OB-M_5_	OB-M_15_	OB-M_20_
Triglycerides (mg/dL)	111 ± 9.6	122 ± 11.4	109 ± 9.3	101 ± 5.4
Cholesterol (mg/dL)	74.2 ± 3.4 ^ab^	83.9 ± 6.3 ^a^	67.4 ± 2.7 ^bc^	63.0 ± 2.3 ^c^
HDL-C (mg/dL)	54.4 ± 2.5 ^b^	63.9 ± 5.0 ^a^	52.7 ± 2.3 ^b^	50.2 ± 2.4 ^b^
LDL-C (mg/dL)	4.8 ± 0.5 ^a^	4.3 ± 0.3 ^ab^	3.1 ± 0.2 ^c^	3.4 ± 0.2 ^bc^
TC/HDL-C	1.37 ± 0.02 ^a^	1.31 ± 0.01 ^ab^	1.30 ± 0.02 ^b^	1.28 ± 0.02 ^b^
Glucose (mg/dL)	250 ± 29.8	277 ± 23.9	276 ± 18.5	251 ± 20.6
FFA (mmol/L)	1.6 ± 0.1	1.8 ± 0.2	1.5 ± 0.1	1.5 ± 0.1
Insulin (μg/L)	1.4 ± 0.3 ^b^	1.8 ± 0.2 ^ab^	2.0 ± 0.1 ^a^	2.2 ± 0.2 ^a^
Leptin (ng/mL)	870 ± 574	1153 ± 562	1243 ± 676	835 ± 571
Adiponectin (ng/mL)	1.2 ± 0.2	1.5 ± 0.2	1.7 ± 0.2	1.5 ± 0.2

Data are expressed as the mean ± SD (*n* = 8/group). Abbreviations: HDL-C, high-density lipoprotein cholesterol; LDL-C, low-density lipoprotein cholesterol; TC, total cholesterol; FFA, free fatty acid. C, 5% *w*/*w* soybean oil; OB-M_5_, 5% *w*/*w* experimental oil mix; OB-M_15_, 15% *w*/*w* experimental oil mix; OB-M_20_, 20% *w*/*w* experimental oil mix. ^a,b,c^ Values with different superscripts significantly differ (*p* < 0.05).

**Table 5:** Liver lipid profiles, activities of hepatic lipogenic and lipolysis enzymes, and adipose tissue lipoprotein lipase (LPL) activities in obese hamsters after the experimental period for eight weeks.

Variables	C	OB-M_5_	OB-M_15_	OB-M_20_
*Liver tissue*				
Liver TG (mg/g liver)	12.6 ± 0.1	12.7 ± 0.2	12.8 ± 0.2	13.6 ± 0.9
Liver TC (mg/g liver)	10.4 ± 1.0	10.4 ± 0.9	10.7 ± 0.6	10.9 ± 1.2
Liver FFA (mmol/g liver)	1.79 ± 0.03 ^d^	1.87 ± 0.03 ^c^	1.97 ± 0.02 ^b^	2.07 ± 0.03 ^a^
FAS (nmole/min/mg protein)	2.51 ± 0.53 ^b^	4.99 ± 0.86 ^a^	6.96 ± 0.93 ^a^	5.64 ± 0.77 ^a^
ACC (nmole/min/mg protein)	2.85 ± 0.65 ^b^	5.13 ± 0.78 ^ab^	8.10 ± 2.05 ^a^	3.40 ± 0.84 ^b^
ACO (nmole/min/mg protein)	0.41 ± 0.01 ^c^	1.05 ± 0.19 ^b^	1.58 ± 0.26 ^a^	1.13 ± 0.26 ^b^
CPT-1 (nmole/min/mg protein)	0.56 ± 0.16	0.44 ± 0.19	0.50 ± 0.03	0.44 ± 0.01
*LPL activities in adipose tissues*				
Epididymal fat (mU/mL)	7.7 ± 1.2	7.5 ± 0.7	7.6 ± 0.8	10.1 ± 0.9
Retroperitoneal fat (mU/mL)	11.0 ± 1.1	10.3 ± 1.0	11.0 ± 1.5	10.5 ± 1.5

Data are expressed as the means ± SD (*n* = 8/group). Abbreviations: TG, triglyceride; TC, total cholesterol; FFA, free fatty acid; FAS, fatty acid synthase; ACC, acetyl-CoA carboxylase; ACO, acyl-CoA oxidase; CPT, carnitine palmitoyltransferase. C: 5% *w*/*w* soybean oil, OB-M_5_:5% *w*/*w* experimental oil mix, OB-M_15_:15% *w*/*w* experimental oil mix, OB-M_20_:20% *w*/*w* experimental oil mix. ^a,b,c,d^ Values with different superscripts significantly differ (*p* < 0.05).
